# Association between novel dietary and lifestyle inflammation indices with risk of breast cancer (BrCa): a case–control study

**DOI:** 10.1186/s12937-022-00766-0

**Published:** 2022-03-02

**Authors:** Mohammad Hassan Sohouli, Mohammad Hadizadeh, Farzaneh Mardali, Vahid Sanati, Elma Izze da Silva Magalhães, Mitra Zarrati

**Affiliations:** 1 Cancer Research Center, Shahid Beheshti University of Medical Sciences, Tehran, Iran; 2 Department of Clinical Nutrition and Dietetics, Faculty of Nutrition and Food Technology, Shahid Beheshti University of Medical Sciences, Tehran, Iran; 3 Department of Nutrition, School of Public Health, Iran University of Medical Sciences, Tehran, Iran; 4 Postgraduate Program in Collective Health, Federal University of Maranhão. São Luís, Maranhão, Brazil

**Keywords:** Breast cancer, Carcinoma, Dietary inflammatory score, Lifestyle

## Abstract

**Background:**

Pro-inflammatory diet and lifestyle factors lead to diseases related to chronically systemic inflammation. We examined the novel dietary/lifestyle indicators related to inflammation such dietary inflammation score (DIS), lifestyle inflammation score (LIS), empirical dietary inflammatory index (EDII) and, risk of *Breast Cancer* (BrCa) in Iranian woman.

**Methods:**

In this hospital-based case–control study, 253 patients with BrCa and 267 non-BrCa controls were enrolled. Food consumption was recorded to calculate the DIS, LIS and EDII using a semi-quantitative Food Frequency Questionnaire (FFQ). We estimated odds ratios (ORs) and, 95% confidence intervals for the association of the inflammatory potential with risk of these cancers using binary logistic regression models modified for the case–control design.

**Results:**

Mean ± SD of age and BMI of the study participants were 47.92 ± 10.33 years and 29.43 ± 5.51 kg/m^2^, respectively. After adjustment for confounders, individuals in highest compared to lowest quartile of DIS and EDII had significantly higher risk of BrCa (DIS: 2.13 (1.15 – 3.92), p-trends: 0.012), EDII: 2.17 (1.12 – 4.22), p-trends: 0.024). However, no significant association was observed for LIS (P-trends: 0.374).

**Conclusion:**

Findings of this study suggested that higher DIS and EDI increased the risk of BrCa, but concerning LIS, further investigation is needed.

## Introduction

One of the most common cancers that affect women worldwide is breast cancer)BrCa) [1]. BrCa is the leading cause of cancer death among women in developing countries and, the second cause of mortality in developed countries [2, 3]. Annually, more than 1.5 million cases of BrCa are diagnosed in women worldwide[4].

Current literature has been reported that preventable risk factors related to lifestyle, including smoking, physical activity (independent from obesity), overweight or obesity, and diet are involved in the pathogenesis of cancer [5–8]. These factors expose the individual to a systemic inflammation that disrupts the immune system and leads the inflammation in the organism [9, 10]. Studies have shown that western diets, characterized by higher intakes of red meat, processed meats, refined grains and, lower intake of vegetable, fruits and antioxidant nutrients, significantly increase inflammation and oxidative stress, as well as adipose tissue and contribute to systemic inflammation, thereby increasing the risk of BrCa [11, 12]. However, despite previous studies suggested a link between specific nutrients/food and inflammation, dietary interactions may modify effects of combined nutrients. So, a new approach based on dietary patterns related to inflammation needs to be used.

In the last decades related dietary indicators such as dietary inflammation index (DII)[13] was evaluated in inflammatory-related diseases such as cancer and diabetes [13–16]. However, this index has many limitations. First, this parameter has been validated in relation to the concentration of various inflammatory factors in the serum level and, secondly, the DII index is based on nutrients that due to different food compositions cannot be accurately estimated. Thus, recently, indicators have been proposed to evaluate the potential of pro-inflammatory diet and lifestyle like dietary inflammation score (DIS), empirical dietary inflammation index (EDII), and lifestyle inflammatory score (LIS) [16–18]. These indexes are based on usual dietary intake and relatively easier and more accurate calculation can be done. In a large cohort study, Byrd et al. shown that higher scores of DIS and LIS were related to higher concentration of hs-CRP [19]. Moreover, it has been shown that the new index regards to lifestyle was correlated to systemic inflammation [18]. In a large cohort study, Byrd et al. reported higher risk for colorectal cancer in patients with a high scores of LIS and DIS [17]. Also, a limited number of other studies have shown a positive association between these inflammatory profiles (especially EDII) and an increased risk of some chronic diseases [20–22]. However, to our knowledge, there are no published studies regarding the relationship between these indices and BrCa.

We assumed that different food communities concerning to dietary patterns and lifestyle exposures could identify inflammatory potential in relation to the risk of BrCa. On the other hand, due to the increasing rate of BrCa as well as the fact that today chronic diseases, especially cancer, have imposed a lot of costs on the health system and due to the fact that so far no study has been conducted to evaluate these dietary indices in patients with BrCa and studies about other cancers are very limited, we carried out this study to evaluate and compare new inflammation-related indices including dietary inflammation score, lifestyle inflammation score and, experimental dietary inflammatory index in BrCa patients to find a critical approach to improving or controlling BrCa.

## Methods

### Participant’s characteristics

This case–control study enrolled 253 BrCa patients and 267 control that had referred to the Hazrat Rasoul Hospital and Taleghani Hospital, Tehran, Iran during the recent years (2019–2020). Inclusion criteria for the case group included the following: 1) Breast cancer confirmed by an oncologist and pathology results; 2) A maximum of 6 months have passed since the diagnosis of BrCa (BrCa patients were newly); 3) Willingness to cooperate in the study; 4) Age older than 18 years and under 65 years; 5) Body mass index 18.5–40 kg/m^2^. Patients with a history of other cancers, hormone-related diseases such as PCOS (polycystic ovary syndrome) or endometriosis and, occurrence of metastasis were excluded from our study. The control group was selected from other departments of the hospital, such as ophthalmology, otolaryngology, dermatology, and aesthetics. The group did not have any history of cancer (benign and malignant), inflammatory disease, and hormone-related diseases. Also, each member of the control group included in the study was examined for inflammation by an internal medicine specialist and laboratory results to ensure the absence of inflammation.

In addition, patients were excluded from the study if they followed a special diet during the last 6 months, had used therapeutic supplements, under-reporting, or over-reporting of energy intake (less than 800 kcal and more than 4200 kcal), and not answering more than 40 items in the food frequency questionnaire (FFQ). Matching between two groups was done based on age. The minimum required sample size was calculated based on the ability to detect an odds ratio (OR) of 2 with a case to control ratio of 1:1, 90% power, effect size of 0.3, a type I error ($\alpha$) rate of 5%, and type II error (α) rate of 10% (The minimum required sample size was 250 participants in each group).

### Non-dietary exposure

The expert interviewer was employed. Using the short form of the International Physical Activity Questionnaire (IPAQ) validated by Vasheghani-Farahani[23], we recorded physical activity levels. Also, the following demographic and behavioral information: age (years), education, age at first pregnancy (years), bra wearing at night (yes or no), smoking (yes or no), supplement intake (yes or no; if yes, complementary information about dose and frequency) and exposure to sunlight during the day (1. Less than 30 min a day, 2. Between 30 and 60 min a day, 3. Between 60 and 120 min a day 4. More than 120 min a day) were recorded from the patients.

The clinical characteristics of participants such as use history of oral contraceptive (month), history of benign breast diseases (yes or no), family history of cancer (yes or no), breast cancer family history (yes or no) was asked. Weight, height, and waist circumference, using Seca® scales (made in Germany, with accuracy of 100 g) and stadiometer (accuracy of 0.5 cm) and non-elastic measuring tape (accuracy of 0.5 cm), respectively, were measured by nutritionist through standardized protocol.

### Dietary Assessment

The dietary intake over the previous year was obtained using a validated food-frequency questionnaire (FFQ) which consisted of 168 food items [24]. The type and standard serving size of usual Iranian food were asked by expert nutritionist. Data relating to the consumption of each item were recorded following the options: daily, 1 time/week, 2–4 times/week, 5–6 times/week, 2–3 times/month and, never. Food intake analysis, including energy intake, macro and micronutrients were calculated by Nutritionist IV software.

### Calculation of dietary inflammation score, lifestyle inflammation score and, empirical dietary inflammatory index

***DIS***: The components of DIS includes 18 food groups and beverages: Leafy greens and cruciferous vegetables, tomatoes, apples and berries, deep yellow or orange vegetables and fruit, other fruits and real fruit juices, other vegetables, legumes, fish, poultry, red and organ meats, processed meats, added sugar, high-fat dairy and low-fat dairy, coffee and tea, nuts, other fats, refined grains and starchy vegetables. The calculation of the score of DIS has been established and validated using the study of Byard et al.[18]. Briefly, equivalent amount of each food group related to the components of DIS as well as frequency of consumption and the amount in the recipe of mixed dish were estimated. The recorded food equivalents were converted to grams. Then, we multiplied the weight of each food item by the respective reported inflammatory value [25]. Finally, we summed all the weighted values together to obtain DIS.

***LIS***: The main components of LIS include four risk factors of smoking, alcohol consumption, physical activity, and body mass index[18]. But, in this study, due to religious and cultural prejudices, we do not include the factor of alcohol consumption. Categories of these variables were described as: smoking (‘current’ and ‘former and never’), physical activity (we categorized participants as those who did not or rarely exercised (light), exercised 1 – 2 times/week (moderate), or exercised ≥ 3 times/week (vigorous), body mass index (normal: 18.5 – 24.99 kg/m^2^; overweight: 25 – 29.99 kg/m^2^ and obese: ≥ 30 kg/m^2^). Then, in order to facilitate comparison, we re-categorized the variables in a dichotomous way and allocated each LIS characteristic its relative weight [18], altogether the weighted values to constitute the LIS.

***EDII***: In order to determine the effect of whole foods on the inflammatory potential, the inflammatory index of the diet was evaluated empirically by Tabung, et al.[16]. Thus, consumption of daily servings of processed meat, red meat, organ meat, other fish, other vegetables, refined grains, high-energy beverages, tomatoes, tea, coffee, dark yellow vegetables, leafy green vegetables, snacks, fruit juice, and pizza are the main components of this index. Then, by multiplying each component to the linked weight and sum all the weighted components and dividing by 1000, the mentioned index could be analyzed.

### Statistical analysis

After examining the normality of the numeric variables, T-Test or Mann–Whitney tests were performed to compare the two groups of case and control based on dummy variables. The chi-square test was used to compare the categorical variables. The risk of breast cancer (OR) and respective 95% confidence interval (95%CI) were estimated by binary logistic regression analysis with adjustment for confounders. Mean of DIS, EDII and LIS were compared between the case and control groups. Scores were categorized into quartiles. For individuals in the highest quartile, dietary inflammation was considered higher than in the first quartile. In the analysis with EDII and DIS, models were adjusted for BMI and age (Model 1); and for waist circumference, energy intake, age at first pregnancy, number of children, abortion history, use of anti-inflammatory drugs and vitamin supplements D (Model 2). In models with LIS, we adjusted for age and waist circumference (Model 1); and for energy intake, age at first pregnancy, number of children, abortion history, use of anti-inflammatory drugs, and vitamin supplements D (Model 2). Statistical analysis was performed using SPSS 20 software. A significance level of 0.05 was considered.

## Results

Mean ± SD of age and BMI of the participants were 47.92 ± 10.33 years and 29.43 ± 5.51 kg/m^2^, respectively.

Table 1 shows the demographic, anthropometric, and lifestyle characteristics among BrCa patients and controls groups. BrCa patients in compared with control individuals significantly had higher waist circumference, first pregnancy age, history of abortion, number of children, and C-reactive protein (CRP), but had lower intake of vitamin D supplementation and anti-inflammatory drugs. Furthermore, no significant differences were observed for other characteristics among cases and controls.

**Table 1:** Demographic anthropometric and lifestyle characteristics of participants in case and control groups

Variables	*Groups, mean (SD)*		*P value*^*a*^
	*Case (n* = *253)*	*Control (n* = *267)*	
***Age, y***	48.91(10.46)	47.13(10.08)	0.062
***BMI ***^***b***^***, kg/m***^***2***^	29.61(4.55)	29.07(5.39)	0.222
***Waist- circumference (cm)***	101.15(96.39)	96.39(13.25)	** < 0.001**
***Physical Activity (Met.h/wk)***	33.18(6.11)	32.70(5.20)	0.336
***Smoking (yes), n (%)***	8 (3.2)	9 (3.4)	0.894
***Marriage age, y***	19.43(5.02)	18.98(4.48)	0.296
***Age at first pregnancy, y***	22.29(5.32)	20.35(4.19)	** < 0.001**
***Child number***	2.92(1.43)	2.54(1.59)	**0.005**
***Abortion history (yes), n (%)***	(37.2)94	(29.2)78	**0.049**
***Breast cancer family history (yes), n (%)***	(5.5)14	(4.5)12	0.594
***Family history of cancer (yes), n (%)***	(26.9)68	(20.7)55	0.097
***History of benign breast diseases (yes), n (%)***	(7.9)20	(5.3)14	0.224
***Inflammatory disease history (yes), n (%)***	(12.6)32	(13.2)35	0.863
***Vitamin D supplement (yes), n (%)***	(14.6)37	(24.3)65	**0.005**
***Ever use of OCP***^***c***^*** (yes), n (%)***	(49.8)126	(56)149	0.156
***Anti-inflammatory drugs use (yes), n (%)***	(10.3)26	(17.7)47	**0.015**
***CRP***^***d***^*** (mg/dl)***	1.87 (0.44)	1.02 (0.32)	**0.008**
***Education, n (%)***			0.518
*Illiterate*	(11.6)29	(9)24	
*Low education*	(46.6)116	(50.4)134	
*Higher education*	(42)105	(40.6)108	
***Exposure to sunlight during the day (min)***			
*Less than 30 min*	(28.5)72	(36)96	0.215
*60–30 min*	(32.4)82	(25.5)68	
*120–60 min*	(17)43	(17.2)46	
*More than 120 min*	(22.1)56	(21.3)57	

^a^ Obtained from ANOVA for continuous variables and Chi-square for Categorical variables; ^b^ BMI: body mass index; ^c^ OCP: oral contraceptive pills; ^d^CRP: C-Reactive Protein

Dietary intakes of study participants across case and control groups are shown in Table 2. Although cases had higher intake of energy, fat, saturated fatty acids, cholesterol, carbohydrates, sodium, folate, and iron than to control group, but they had lower intake of monounsaturated fatty acids, potassium, phosphorus, calcium, and micronutrients antioxidants such as zinc, magnesium and vitamins E, C and D.

**Table 2:** Dietary intakes of study participants across case and control groups

	***Groups, mean (SD)***		
	*Case (n* = *253)*	*Control (n* = *267)*	***P value***^***a***^
Energy (Kcal/d)	2753.45(798.02)	2464.1(607.43)	** < 0.001**
Carbohydrate (g/d)	56.18(7.47)	54.24 (7.04)	**0.002**
Protein (g/d)	13.02 (2.15)	13.03 (2.13)	0.984
Fat (g/d)	35.11(6.75)	33.14(7.61)	**0.002**
SFA^b^ (g/d)	32.92 (11.26)	29.20(10.53)	** < 0.001**
MUFA^c^ (g/d)	32.29 (13.29)	37.24 (15.97)	** < 0.001**
PUFA^d^ (g/d)	20.48 (10.35)	24.49 (13.29)	** < 0.001**
Cholesterol (mg/d)	293.52(135.55)	261.88(139.27)	**0.009**
Fibre(g/d)	37.96 (19.28)	39.89 (18.58)	0.247
Sodium (mg/d)	4740.74(1811.95)	4307.06(1898.50)	**0.008**
Potassium (mg/d)	3766.23(1224.29)	4297.22 (1261.12)	** < 0.001**
Phosphor (mg/d)	1482.87 (492.60)	1617.48 (485.35)	**0.002**
Iron (mg/d)	20.28 (9.96)	16.34 (6.06)	** < 0.001**
Calcium (mg/d)	1215.79 (463.90)	1335.27 (458.76)	**0.003**
Magnesium (mg/d)	370.06(119.89)	402.91 (133.15)	**0.003**
Zinc(mg/d)	11.76 (3.82)	12.95 (4.05)	**0.001**
Vitamin C(mg/d*)*	159.16(89.15)	197.87 (78.89)	** < 0.001**
Folate (mcg/d)	485.57 (168.28)	455.20 (163.07)	**0.037**
Vitamin B12 (mcg/d)	5.53 (3.87)	6.70 (4.53)	**0.002**
Vitamin E (mg/d)	17.64 (13.16)	23.59 (17.54)	** < 0.001**
Vitamin D (mcg/d)	2.04 (3.44)	2.7 (3.06)	**0.012**

^a^Obtained from ANOVA; ^b^SFA: saturated fatty acid; ^c^MUFA: monounsaturated fatty acid; ^d^PUFA: polyunsaturated fatty acid

Tables 3 and 4 show the indexes of the components among subjects of case and control groups. Regarding EDII components, higher intake of processed meat, high-energy beverages, coffee, and lower intake of other fish, other vegetables, tomatoes, dark yellow vegetables, and leafy green vegetables were observed in the cases compared to the controls. Furthermore, dietary intake of leafy greens and cruciferous vegetables, tomatoes, deep yellow or orange vegetables and fruit, other fruits and real fruit juices, other vegetables, legumes, fish, low-fat dairy, and nuts was significantly lower in patients with BrCa compared to controls. However, dietary intake of apples and berries, processed meats, added sugars, refined grains, and starchy vegetables was significantly higher in these patients. Also, the proportion of individuals with overweight or obesity, as a component of LIS, was higher among BrCa patients than to controls.

**Table 3:** Dietary intakes of EDII (Empirical Dietary Inflammatory Index) components of the study participants

	***Groups, mean (SD)***		***P*-value** ^a^
	*Case (n* = *253)*	*Control (n* = *267)*	
**EDII component**			
**EDII score**	1.98 (0.96)	1.02 (0.69)	**0.016**
Processed meat (serving/d)	0.04 (0.06)	0.01 (0.03)	** < 0.001**
Red meat (serving/d)	0.16 (0.15)	0.16(0.12)	0.628
Organ meat(serving/d)	0.03 (0.07)	0.04 (0.08)	0.617
Other fish(serving/d)	0.10 (0.15)	0.12 (0.14)	**0.029**
Other vegetables(serving/d)	1.85 (1.14)	2.20 (1.16)	**0.001**
Refined grains(serving/d)	6.14 (3.40)	5.66 (3.25)	0.104
High-energy beverages(serving/d)	0.18 (0.33)	0.12 (0.28)	**0.038**
Tomatoes(serving/d)	0.80 (0.69)	0.96 (0.66)	**0.010**
Tea(serving/d)	2.98 (2.37)	3.24 (2.24)	0.206
Coffee(serving/d)	0.05 (0.19)	0.02 (0.07)	**0.014**
Dark yellow vegetables(serving/d)	0.25 (0.28)	0.34 (0.34)	**0.001**
Leafy green vegetables(serving/d)	0.30 (0.25)	0.39 (0.38)	**0.002**
Snacks(serving/d)	0.28 (0.64)	0.35 (0.85)	0.333
Fruit juice(serving/d)	0.05 (0.12)	0.03 (0.09)	0.085
Pizza(serving/d)	0.03 (0.05)	0.02 (0.05)	0.281

^a^ Obtained from ANOVA

**Table 4:** LIS (Lifestyle Inflammation Score) and DIS (Dietary Inflammation Score*) components* among breast cancer and control groups

	***Groups, mean (SD)***		***P*-value**
	*Case (n* = *253)*	*Control (n* = *267)*	
**LIS Component**			
**LIS score**	0.94 (0.54)	0.85 (0.62)	0.048
Current smoker, *N*(%)	(3.2)8	(3.4)9	0.894
Physical activity categories			0.941
Moderately physically active, N(%)	142 (56.1)	151 (56.6)	
Heavily physically active, N(%)	25 (9.9)	24 (9.6)	
BMI categories			**0.002**
Overweight BMI, N(%)	112 (44.3)	93 (34.8)	
Obese BMI, N(%)	109 (43.1)	109 (40.8)	
**DIS component**			
**DIS score**	-0.15 (1.11)	0.06 (0.19)	**0.002**
Leafy greens and Cruciferous vegetables(g/d)	19.3 (16.2)	25.5 (22.8)	** < 0.001**
Tomatoes(g/d)	100.9 (85.3)	120.4 (82.3)	**0.008**
Apples and berries(g/d)	50.1 (54.0)	40.4 (35.9)	**0.015**
Deep yellow or orange Vegetables and fruit(g/d)	69.5 (62.0)	102.9 (69.5)	** < 0.001**
Other fruits and real fruit juices(g/d)	268.4 (173.1)	342.0 (157.9)	** < 0.001**
Other vegetables(g/d)	71.3 (44.4)	89.6 (42.4)	** < 0.001**
Legumes(g/d)	24.4 (21.8)	36.7 (28.9)	** < 0.001**
Fish(g/d)	11.3 (17.8)	14.6 (16.4)	**0.029**
Poultry(g/d)	27.9 (25.8)	28.3 (20.9)	0.861
Red and organ meats(g/d)	28.3 (24.1)	29.6 (21.3)	0.506
Processed meats(g/d)	6.44 (9.87)	2.50 (4.25)	** < 0.001**
Added sugars(g/d)	79.4 (90.8)	64.7 (78.5)	**0.048**
High-fat dairy(g/d)	98.6 (95.4)	88.3 (111.8)	0.260
Low-fat dairy(g/d)	260.4 (200.4)	311.9 (191.7)	**0.003**
Coffee and tea(g/d)	729.3 (569.5)	783.3 (540.2)	0.267
Nuts(g/d)	8.6 (10.7)	12.6 (12.9)	** < 0.001**
Other fats(g/d)	36.9 (25.4)	39.9 (30.7)	0.214
Refined grains and Starchy vegetables(g/d)	362.8 (197.2)	326.5 (172.1)	**0.025**

Odds ratio (OR) and 95% confidence interval (95%CI) for breast cancer based on quartiles of inflammatory indices were presented in Table 5. In crude model and after adjusting for age and BMI (Model 1), subjects in the highest *versus* lowest quartile of EDII showed no statically significant risk of BrCa. However, when adjusted for waist circumference, energy intake, age at first pregnancy, number of children, abortion history, use of anti-inflammatory drugs and vitamin supplements D (Model 2), those in the highest EDII quartile were more likely to have BrCa compared with those in the lowest quartile (OR:2.17, 95%CI: 1.12 – 4.22; *P* for trend = 0.024).

**Table 5:** *Odds ratio (OR) and 95% confidence interval (CI)*) *for breast cancer based on Quartiles of inflammatory indices*

	**Quartiles of scores**				***P* for trend**
	**Q1**	**Q2**	**Q3**	**Q4**	
**DIS**					
^a^Case/Total	50 / 130	53 / 130	65 / 130	85 / 130	
Crude model	1.00 (Ref)	0.84 (0.48 – 1.47)	1.09 (0.62 – 1.91)	2.56 (1.48 – 4.44)	< 0.001
Model 1^a^	1.00 (Ref)	0.88 (0.50 – 1.54)	1.19 (0.67 – 2.10)	2.67 (1.53 – 4.65)	< 0.001
Model 2^e^	1.00 (Ref)	0.82 (0.44 – 1.50)	0.96 (0.51 – 1.79)	2.13 (1.15 – 3.92)	0.012
**EDII**					
^a^Case/Total	53 / 130	66 / 130	68 / 130	66 / 130	
Crude model	1.00 (Ref)	1.23 (0.70 – 2.16)	1.29 (0.74 – 2.25)	1.39 (0.80 – 2.42)	0.239
Model 1^a^	1.00 (Ref)	1.28 (0.73 – 2.26)	1.35(0.77 – 2.37)	1.49 (0.85– 2.62)	0.166
Model 2^†^	1.00 (Ref)	1.44 (0.77– 2.67)	1.57 (0.84 – 2.92)	2.17 (1.12 – 4.22)	0.024
**LIS**					
^a^Case/Total	44 / 130	64 / 130	98 / 130	47 / 130	
Crude model	1.00 (Ref)	1.72 (0.94– 3.15)	1.79 (1.04 – 3.06)	1.47 (0.79 – 2.73)	0.169
Model 1^d^	1.00 (Ref)	1.31 (0.69 – 2.48)	1.10 (0.59 – 2.04)	0.74 (0.35 – 1.57)	0.387
Model 2^f^	1.00 (Ref)	1.24 (0.62 – 2.48)	1.07 (0.55 – 2.07)	0.70 (0.31 – 1.55)	0.374

^***a***^Model 1: adjusted for age and BMI

^***b***^*** Case:**** Breast cancer patients, ****Total:**** Total subjects (case and control subjects)*

^***c***^*** Binary logistic regression was used to obtain OR and 95% CI***

^d^Model 2: Waist circumference, energy, age at first pregnancy, number of children, history of abortion, use of anti-inflammatory drugs and vitamin supplements D

^e^ Model 1: adjusted for age and waist circumference

^f^Model 2: Energy, first pregnancy age, number of children, history of abortion, use of anti-inflammatory drugs and vitamin supplements D

In the crude model, the risk of BrCa was higher in individuals in the highest DIS quartiles compared to those in the lowest quartile (OR: 2.56, 95%CI: 1.48 – 4.44; p for trend < 0.001). Furthermore, after adjustment for confounders (Model 1 and 2), in the highest versus lowest DIS quartiles, the risk of BrCa remained significant (OR: 2.67, 95%CI: 1.53 – 4.65; P for trend < 0.001 and OR: 2.13, 95%CI: 1.15 – 3.92; P for trend = 0.012, respectively).

However, no significant association was observed between LIS and risk of BrCa, neither in the crude model nor adjusted models.

## Discussion

This case–control study in Iranian women showed that higher EDII and DIS scores significantly increased risk of BrCa, but in crude and adjusted models, there was null association between LIS and risk of BrCa. Also, the results of further analysis on the EDII and DIS indices showed that women in the highest quartile after adjusting for potential confounders in fully adjusted model than to the lowest quartile, had a higher risk of BrCa.

The EDII was developed through a data driven approach to identify food groups most associated with plasma inflammation biomarkers in a subset of the Nurse’s Health Study cohort. The limitations of this index included such things as reproducibility, generalizability, and assumptions that human experiences were strongly combined with. While the DIS and LIS indexes, based on the FFQ lifestyle questionnaire responses in various populations, was determined by quantifying different associations of aggregates of food groups and of lifestyle exposures with a panel of circulating biomarkers of systemic inflammation and a hypothesis-based method (instead of an experimental, data-driven one) has been used to improve further epidemiological research into the role of diet in inflammation and the cause of inflammatory diseases. Therefore, in this study, we tried to examine and compare these indices related to inflammation with the risk of BrCa.

To the best author knowledge, the current study is pioneer in evaluation of potential inflammatory indexes associated with lifestyle, diet, and empirical index e.g., DIS, LIS, EDII, in BrCa patients. Both BrCa and inflammatory biomarkers are influenced by dietary components and are closely related to each other. However, there are limited studies which evaluated the association between DIS and LIS with risk of diseases [17–19].

To date, no study has evaluated the association between EDII and DIS in BrCa patients, but Byrd et_al [17] as mentioned previously, indicated that individuals in higher DIS quintile had higher risk of colorectal cancer (HR: 1.27,( 1.19 to 1.35) P-trend < 0.001). Also, in a large case–control study in Australian women [26] had been reported that EDII scores were significantly higher in patients with ovarian cancer (OR EDII score Q4 *vs.* Q1 = 1.391; *p*-trend = 0.002). Relating to EDII, there are several studies in agree with our findings which demonstrated that higher EDII was related to higher potential of inflammation. For example, Tabung et al. [27] in 2017, showed that individuals in highest EDII quintile had higher concentration of CRP and IL-6 and TNF-a than those in the lowest quintile. Another multi-ethnic study showed that higher scores of EDII were associated with increased IL-6, CRP in postmenopausal women [21]. Also, findings from Tehran Lipid and Glucose Study [20] indicated that components of metabolic syndrome such as abdominal obesity, were related to higher EDII scores.

Researchers have studied the relationship between different pro/anti-inflammation dietary patterns and BrCa risk in several studies. Western dietary pattern with high content intake of processed red meat, high-fat dairy products, sweets, refined grains, and sauces contributed elevation in serum markers such as TNF-a and CRP [28]. Castelló in a large case–control study, had been showed that both premenopausal and postmenopausal women with high adherence to western dietary pattern were at high risk for BrCa (OR: 1.68 (1.02;-2.79), (95%CI) and OR: 1.48 (1.07; 2.05(95%CI), respectively) [29]. On the other hand, previous studies have shown that the Mediterranean diet, which is found to be high source of antioxidants, omega-3, fish, fiber, vegetables and fruits, olive oil, legumes, and nuts reduced the concentration of inflammatory markers [30]. In a systematic review conducted by Turati et al., it was shown that in European women who were following to Mediterranean-style Dietary pattern, the risk of breast cancer was lower (OR: 0.82 (0.71–0.95) p-trend: 0.008) [31]. Results of LIS revealed that women with BrCa had higher weight than non BrCa patients. It seems that in obese or overweight cases, complex mechanisms appear to stimulate hormone-receptor-negative tumors, and increase in TNF-α, IL-6 [20, 21]. However, the results of the present study do not support that association between LIS and BrCa risk. In line with our study, records from the NIH-AARP Diet and Health Study [22] showed that factors related to lifestyle like smoking, body mass index, and anti-inflammatory drugs as well as lifestyles factors had null association with pancreatic cancer.

While in REGARDS cohort study by Byrd [18] has been demonstrated that individuals in highest LIS quintile compare to healthy individuals resulted to 4.29 times increasing in CRP concentration in ischemic stroke patients. We suggest that this discrepancy in the results of our study and NIH-AARP Diet and Health cohort [22] with cohort REGARDS [18] may be due to the lack of adjustments for other supplements involved in inflammation such as vitamins E, C and A. So, we strongly recommend that other supplements be considered in future LIS analysis. We also propose that the severity of inflammation influences the association of factors related to lifestyle and disease risk.

Because breast cancer is considered a low grade inflammation relative to stroke, thus no significant results were observed [32]. The role of inflammation in the pathogenesis of breast cancer from tumor genesis to progression appears to be unclear and is not fully understood. One of the suggested mechanism is the imbalance of the omega-3 to omega-6 ratio through mediators that produce inflammatory cytokines such as prostaglandin E2 [33]. Typically, in areas far from the sea, omega-3 is significantly reduced as fish intake decreases. In a recent meta-analysis, it was established that processed meat seems to be related with increased risk of BrCa due to high amount of nitrite and nitrate, saturated fatty acids, cholesterol, and heme iron [34]. But, in a plausible pathway, dietary pro-inflammations in pathogenesis of BrCa are related to insulin-like growth factor (IGF-1), and so cell proliferation and apoptosis will be stimulated by IGF-1 mediation [35].

Our study, also showed that BrCa patients had lower intakes of dietary antioxidant compounds such as zinc, magnesium, and vitamins E, C, and D, which are components of the DII and EDII. Previous studies have examined the effect of antioxidant compounds on the immune system and chronic diseases such as cancer [36]. In line with our study, for example, in a prospective cohort study [15], it was shown that the total antioxidant capacity of a diet containing beta-carotene (like tomatoes), flavonoids (fruits and vegetables), vitamins A, E, and selenium were associated with a lower risk of breast cancer [37]. The anti-cancer effects of antioxidants in interaction with IGF-1 signaling and pathway involving target-of-rapamycin (TOR) may control cell growth [38]. We also showed that consumption of refined grains was associated with higher EDII scores. A randomized clinical trial in Denmark [39] reported that consumption of whole grains compared to refined grains reduced biomarkers in systemic inflammation as such IL6 and CRP, providing strong evidence for the effects of anti-inflammatory diet and corroborating with our study.

Some relevant findings deserve to be highlighted in this study. Firstly, our research is the first evaluation of dietary and lifestyle inflammation indices among breast cancer patients, which tests the inflammatory potential with a combination of lifestyle and dietary. Secondly, we tried to find a correct and comprehensive approach to assessing inflammation in BrCa by combining the interactions between lifestyle and dietary factors**.** One of the major limitations of the research, as in other case–control studies, is the “memory” factor, which implies a possible measurement error, since the collection of data on lifestyle and diet was performed by self-report. Besides, we could not measure a panel of inflammatory biomarkers in the study participants.

## Conclusion

Our findings, along with previous literature, suggest that diets and lifestyles that reflect pro-inflammatory factors, especially if taken in combination by individuals, may be associated with a higher risk of BrCa. Future research into the association of diet-related inflammation and lifestyle with BrCa incidence and survival using the new DIS and LIS could support our findings.

## Data Availability

Data available on request due to privacy/ethical restrictions.
